# New Era Dawning under State Auspices: Nurses' Pensions: The Poor-Law Nurse

**Published:** 1919-05-10

**Authors:** 


### NURSING AND MIDWIFERY PROBLEMS.

### New Era Dawning under State Auspices: Nurses' Pensions: The Poor-Law Nurse.

THE Conference held in connection with the Nursing Exhibition in London continued to be well attended on Thursday and Friday. Some of the papers were followed by animated discussions, in which the speakers displayed wide knowledge of the subjects under consideration.

### State Aid for Midwifery.

A scheme for a "State-aided Midwifery Service" was outlined by Miss Emily Ford, Secretary of the Association for Promoting the Training and Supply of Midwives. She said the reason why only 7,000 midwives are practising out of 30,000 who are qualified was that the remuneration is inadequate. To live by midwifery a woman must earn not less than £150 in the towns and £120 in the country. A midwife could attend only about 120 cases in the year; therefore, to reach this income she should receive 25s. for each patient. This amount should be guaranteed by the State. To obtain the money from the local rates would not be satisfactory, though the scheme might be administered by the local authorities. The cost to the State was estimated at £1,000,000 a year. Inspection of midwives was not what it ought to be. Only women should be employed in this capacity, and there should be a high standard of qualifications.

In the discussion, the principal grievance ventilated was that while midwives must have a certificate to practise, inspectors of midwives needed no certificate.

### Admirers of the Poor-Law Nurse.

The Poor-Law nurse has a warm admirer in the Rev. A. Lombardini, chaplain of St. Elizabeth's, Kensington, who read a paper touching upon what he called the "Poor-Law Stigma." Referring to an attitude of aloofness he had remarked at social gatherings on the part of nurses from the big general hospitals towards nurses from Poor-Law infirmaries, he said it was begotten of lack of knowledge and inherent prejudice. An infirmary which was recognised as a training-school was the same as a hospital in which the best medical skill is employed. "Take a nurse trained in an approved infirmary training-school, let her start level with a hospital-trained nurse, and watch the result. The infirmary nurse would not be last." Infirmary-trained nurses had won golden opinions from surgeons and matrons for their services during the war. There were, of course, infirmaries which from their size could not be training-schools, but he looked forward to the time when these smaller institutions would be incorporated into a large one under one matron. Mr. Lombardini put in a plea for more recreation facilities and off-duty time for nurses, who were human beings, and not machines. In his opinion, a great time is dawning for the nursing profession; the College of Nursing, with its admirable chairman and practical Council, was providing impetus to a great movement of emancipation. As a supporter of all that the College stands for, he said, "Stand to your guns, and do not be baulked of your victory. Let your policy be bold, and do not withdraw from a situation which is clearly to your advantage. Do not lose the initiative; let no one forestall you." As to Nurses' Day, it was the tribute of a grateful nation to a noble body of women, and he disagreed with those people who were unhappy because they thought this National Tribute was charity and beneath the dignity of a profession to accept. Finally, he said to nurses, "You are going to have good pay, good hours, and healthy surroundings; be patient and loyal."

Miss A. C. GIBSON (who was in the chair) said when she first came to London she noticed the atmosphere of aloofness referred to by Mr. Lombardini, and discovered that it was due, first, to the fact that she was a provincial, and, secondly, because she happened to be matron of a Poor-Law infirmary. But she was proud to have been a Poor-Law matron, and had the greatest admiration for the infirmary nurse. If she were ill she would have one of her own nurses if she could get one, but if not she would send for another Poor-Law nurse.

Mrs. LATTER said she did not think the prejudice in the hospital world against the Poor-Law nurse existed to-day; she imagined it was a thing of the past. (Signs of dissent.) As one who had been in charge of a Red Cross hospital, she could bear testimony to the admirable work done by Poor-Law nurses. She found the Poor-Law nurse could more than hold her own with hospital-trained nurses, who came fortified with a wonderful array of certificates.

### To Centralise Country Infirmaries.

Mrs. LATTER dealt with the subject of nursing in small country workhouses, and propounded a centralisation scheme. Very little progress had been made in country places. She wanted to see treatment in infirmaries which were set apart from the workhouse. She would group the workhouses in a given area, say a radius of twenty miles, and send the infirm and sick to a central infirmary, which might have from 1,000 to 2,000 beds. Counties should be divided into four parts—north, south, east, and west—and each part provided with an infirmary well equipped for scientific treatment and research, and each acting as a medical training-school. The greater transport facilities promised would serve to link up the villages. As to the expense, she thought it would prove cheaper to run one large hospital than many small establishments as now. Anyhow, steps would have to be taken in the near future, and perhaps the Minister of Health would have something to say on the subject.

### Grants Coming for District Nursing.

THE COUNTESS OF MARCH, Hon. Secretary Q.V.J.I., dealt with "District Nursing Under the Ministry of Health." Great things, she said, were expected of the new Ministry, but whether they would be realised she did not know. She had had two conversations with those concerned in the formation of the Ministry, and they had given her to understand that district nursing would become a much more valuable asset to the country. If district nurses were ready to do what was asked they would receive great help in the way of grants. In the country districts, at any rate, district nurses would probably be expected to act as school nurses. After all, they had always looked after the children, and whether they did so in the schools or the homes really did not matter. If district nurses adapted themselves to the new circumstances there would be no danger of being snuffed out, as some seemed to fear. Health visitors had been a source of anxiety to district nurses, and she was

**Future of Nursing Discussed**—(*continued*).

an advocate of all being placed under the same authority and pulling together. That, she was assured from a high source, would be brought about; all would be placed under the same bodies and on the same level. District nurses must be ready to fall in with new ideas, and she believed the Ministry of Health would not fail them. She was a strong believer in co-ordination; it was not fair that the mother's home should be invaded by so many officials as at present.

### Hospital Nurses in a Groove.

Miss Masters, Superintendent Paddington D.N.A., speaking on "Preventive Nursing: A Wider Outlook for Nurses," said nurses in hospitals had no idea what district nursing means. The schools provided for only certain classes of training—medical and surgical were the chief. But the old order of training must pass away; nurses must engage in more special work before passing their final examination. For instance, it was difficult for hospital nurses who were not accustomed to make reports to give a detailed account of their cases; they must be trained to do it. They should also know more about the nursing of children, have a knowledge of hygiene and sanitation—subjects which are not taught in the hospitals—and gain an inkling of the treatment of infectious diseases, as well as study tuberculosis nursing before getting their final certificate. Unless nurses did make themselves acquainted with these matters there was danger of their being supplanted when the Ministry of Health comes along. Nurses in hospitals must get out of the groove: they saw patients under the best conditions in hospitals, and had not the least idea of the conditions that obtained in some of the people's homes; hence the necessity for the study of social conditions.

### Why Not Pensions for Nurses?

Lady Bathurst made interesting suggestions in a paper on "Pensions for Nurses." In any pensions project that might be launched she hoped there would be one fund only, which would embrace not only matrons, sisters, staff nurses, and probationers, but all duly registered private nurses and district and village nurses. Any scheme should include disablement pay as well as pension. Would it not be possible for the Nation's Fund to form a benefit society, to which nurses could contribute instead of to the National Insurance Fund? At the beginning a grateful people would contribute something, and in a few years the scheme might be self-supporting. She understood that the ordinary nurse's working life extended only to fifteen or sixteen years, and it was deplorable that at present there was little or nothing for her to fall back upon. Pensions should be payable at fifty-five or fifty-six years of age. Many hospitals were, she was glad to know, raising the nurses' pay, but even now it was better to, say, mend roads in a leisurely manner than to take charge of human life. (Hear, hear.)

### An Advocate of Eight Hours' System.

Miss Edmondson, matron, Aberdeen Royal Infirmary, delighted the audience by her outspoken remarks on "The Present Conditions of the Nursing World." She was ready to acknowledge the work done for nurses before the College of Nursing came into the field, but with the advent of this body there came a fresh breeze, almost amounting to a gale. She was strongly in favour of co-operation and affiliation, and was an advocate of preparatory schools for nurses, who would enter at seventeen or eighteen years of age as the beginning to a more embracing and extended course of training. More and more nurses would be wanted. On the question of hours, she did not see how, for probationers and staff nurses, the eight-hours' system could not be made to work. Where it had failed, it was because the nurses employed were too few. The main obstacle raised was that there was no accommodation for more nurses. To that she replied, "Billet them out." That plan had been tried in some parts of Scotland, and the nurses were never happier, because it gave them change and chances of recreation. Nurses wanted recreation and more freedom. There were matrons who would have this world an Adamless Eden, and yet these very same matrons objected to a woman house-surgeon! (Laughter). Staff nurses should be treated as if they were gifted with intelligence, and sisters ought to have more scope and be regarded as sectional matrons. As to registration, let all in the nursing profession drop bickerings and personalities and work for the common good. There was plenty to do.

